# Individualized hemodialysis: Is similar hemodialysis adequacy possible using less water?

**DOI:** 10.55730/1300-0144.5756

**Published:** 2023-10-10

**Authors:** Ali AKTAŞ, Hakan ÖZER, İsmail BALOĞLU, Halil Zeki TONBUL, İbrahim GÜNEY, Nedim Yılmaz SELÇUK

**Affiliations:** 1 Department of Internal Medicine, Faculty of Meram Medicine, Necmettin Erbakan University, Konya, Turkiye; 2 Division of Nephrology, Department of Internal Medicine, Konya City Hospital, Konya, Turkiye; 3 Division of Nephrology, Department of Internal Medicine, Faculty of Meram Medicine, Necmettin Erbakan University, Konya, Turkiye

**Keywords:** Hemodialysis, dialysis adequacy, dialysate flow rate, online Kt/V

## Abstract

**Background and aim:**

There are over 60,000 hemodialysis (HD) patients in Türkiye, and the number of patients is increasing yearly. Dialysate flow rate (Qd) is a factor in HD adequacy. Approximately 150 L of water are consumed per session to prepare the dialysate. We aimed to investigate whether HD effectiveness can be achieved at a low Qd in different patient groups for the purpose of saving water.

**Materials and methods:**

This prospective study included 81 HD patients from 2 centers. The patients underwent an aggregate total of 486 HD sessions, including 3 sessions at a Qd of 500 mL/min and 3 sessions at a Qd of 300 mL/min for each patient. We used online Kt/V readings recorded at the end of each dialysis session to compare the effectiveness of these 2 types of HD session performed at a different Qd.

**Results:**

The online Kt/V readings were similar between the standard (500) and low (300) Qd HD (1.51 ± 0.41 and 1.49 ± 0.44, respectively, p = 0.069). In the subgroup analyses, men had higher online Kt/V values at the standard Qd compared to the low Qd (1.35 ± 0.30 and 1.30 ± 0.32, respectively, p = 0.019), but the Kt/V values were not different for women. While the low Qd did not reduce online Kt/V in patients using small surface area dialysis membranes (1.75 ± 0.35 for 300 Qd and 1.75 ± 0.32 for 500 Qd, p = 0.931), it was associated with reduced online Kt/V in patients using large surface area dialysis membranes (1.12 ± 0.25 for 300 Qd and 1.17 ± 0.24 for 500 Qd, p = 0.006). The low Qd did not result in differences in online Kt/V among low-weight patients. However, online Kt/V values were better with the standard Qd in patients weighing 65 kg and above.

**Conclusion:**

In our study, dialysis adequacy at a reduced dialysate flow was not inferior for women, patients with low body weight, or patients using small surface area membranes. Individualized HD at a reduced Qd of 300 mL/min in eligible patients can save 48 L of water per HD session and an average of 7500 L of water per year.

## 1. Introduction

Hemodialysis (HD) is the most common renal replacement therapy used for patients with end-stage renal disease (ESRD). Out of approximately 3 million ESRD patients globally, 2 million live on HD [1]. One of the most critical factors affecting the life span and the quality of life of HD patients is dialysis adequacy. Dialysis adequacy is affected by many variables, such as the blood and dialysate flow rate (Qd), the volume of ultrafiltration, the dialyzer membrane surface area, the mass transfer coefficient for solutes, and the dialysis duration. The effect of Qd on dialysis clearance is limited by the blood flow rate and the dialyzer membrane surface area, with a Qd of 500 mL/min accepted as the standard since the 1960s [2]. Dialysis fluid is produced by treating water with different processes for use in HD. With a standard HD prescription (a 4-hour session at a Qd of 500 mL/min 3 times a week), a patient consumes an average of 150 L of water per session and approximately 20,000 L of water per year [3,4].

The most commonly used parameters to evaluate dialysis adequacy are urea reduction ratio (URR) and Kt/V_urea_. The US National Kidney Foundation’s Kidney Disease Outcomes Quality Initiative recommends a target Kt/V of 1.2 and a URR of over 70% [5]. The alternative method for calculating Kt/V is based on measuring the difference between the conductivity of the dialysis fluid entering and leaving the dialyzer and the difference in the electrolyte concentrations of these fluids. Online Kt/V is a practical method to assess dialysis adequacy by calculating the ionic clearance of sodium [6,7].

Drought is one of the most critical problems globally, also affecting Türkiye. Of the world’s population, 40% do not have access to clean water due to the uneven distribution of water resources. It is estimated that by 2025, 1.8 billion people will live in areas with permanent water scarcity [8].

At the end of 2020, there were over 60,000 HD patients in Türkiye, and the number of patients is increasing yearly. The significance of the drought problem and the significant volume of water consumed for HD have led researchers to search for new methods to reduce water use in HD. In this study, we aimed to investigate the effect of a reduced Qd at 300 mL/min compared to 500 mL/min on dialysis adequacy while keeping the other variables of HD constant.

## 2. Materials and methods

This prospective study included 81 chronic HD patients (40 men, 49.65 ± 16.64 years) from both a medical faculty hospital and a city hospital in a province of Türkiye. These patients were followed up for an aggregate total of 486 HD sessions. The effects of standard (500 mL/min) and low (300 mL/min) dialysate flow rates on HD adequacy were investigated using online clearance monitoring (OCM).

### 2.1. Inclusion and exclusion criteria

Patients over the age of 18, who had been receiving HD treatment 3 days a week for at least 6 months, receiving HD treatment on machines that can measure Kt/V online (Fresenius 4008 and B. Braun Dialog+), and whose Kt/V was 1.2 and above, were included.

Patients under the age of 18; receiving HD treatment with emergency HD indications; having a Kt/V value below 1.2 in the last 3 measurements; pregnant patients; and patients with severe heart failure, pulmonary pathology, or liver disease were excluded.

### 2.2. Study protocol

In the first week, all patients underwent HD on their routine HD days at a dialysate flow rate of 300 mL/min (low flow dialysate) in all 3 sessions. The following week, the same patients underwent HD at a dialysate flow rate of 500 mL/min (standard flow dialysate) in all 3 sessions. Dialysis time (4 h), blood flow rate (300 mL/min), and dialysate temperature (36.5 °C) were kept constant in all sessions. Fresenius Medical Care, Baxter, and Clearum Medtronic branded dialyzers were used. The ultrafiltration coefficient values of the dialyzers ranged from 14 to 22 mL/h/mmHg, while the urea transfer-area coefficient values were >700 mL/min. Dialysis adequacy was determined using online Kt/V at the end of the fourth hour of each session for each patient. For online Kt/V, the sodium conductivity-based clearance calculation method was used with OCM. OCM with electrical conductance was performed using a Fresenius 4008 or B. Braun Dialog+ dialysis machine upon the initiation of the HD session. Each patient had their HD treatments consistently with the same type of machine. The dialysis machines used electrical conductivity to evaluate urea variations based on sodium changes and displayed HD adequacy as an online Kt/V value on a monitor with a urea clearance indicator. The ultrafiltration volume, arterial and venous pressure, inlet and outlet blood pressures, and blood pressure variability were recorded in all sessions.

### 2.3. Ethical considerations

The ethics committee of Necmettin Erbakan University approved this prospective study with the decision number 2022/3591 on 07.01.2022. All patients meeting the eligibility criteria signed informed written consent.

### 2.4. Statistical analysis

The data were analyzed using SPSS version 21.0. For the descriptive analyses, frequency data were presented using numbers (n) and percentages (%), and numerical data were presented using mean ± standard deviation and 95% confidence interval (CI). The conformity of the numerical data to a normal distribution was evaluated using the Kolmogorov–Smirnov test, Shapiro–Wilk test, and skewness and kurtosis values. Normally distributed numerical data between 2 dependent groups were examined using the paired samples t-test (dependent t-test). Non-normally distributed data between 2 dependent groups were examined using the Wilcoxon test. The results were evaluated within a 95% confidence interval. A p value of <0.05 was considered significant.

## 3. Results

This study included 81 chronic HD patients (40 men, 49.65 ± 16.64 years). The most common comorbidity was hypertension in 63% of the patients. The patients’ mean weight was 64.14 ± 15.50 kg and the mean BMI was 23.86 ± 5.17 kg/m^2^. Table 1 lists the demographic and clinical characteristics of the patients.

For all 81 patients in the study, HD adequacy evaluated using online Kt/V readings was not significantly different between the standard and low Qd HD (1.51 ± 0.41 and 1.49 ± 0.44, respectively, p = 0.069). The ultrafiltration volume was higher at the standard Qd compared to the low Qd (p = 0.001) (Table 2).

The examination of the effect of the Qd on online Kt/V readings by gender revealed no significant differences in HD clearance between the standard and low Qd values for women. However, the online Kt/V readings were higher at the standard Qd than the low Qd for men (1.35 ± 0.30 and 1.30 ± 0.32, respectively, p = 0.019) (Table 2).

The patients’ mean weight was 64.14 ± 15.50. The effect of the Qd on dialysis adequacy was compared between patients weighing below (n = 51) and above (n = 30) the study’s mean body weight. The low Qd in low-weight patients did not cause a difference in HD adequacy calculated with online Kt/V. However, online Kt/V readings were higher at the standard Qd than at the low Qd for patients weighing 65 kg and above (1.25 ± 0.32 and 1.22 ± 0.34, respectively, p = 0.028).

The analysis based on type of dialyzer membrane revealed no significant effect of the low Qd on online Kt/V (1.75 ± 0.35 for 300 Qd and 1.75 ± 0.32 for 500 Qd, p = 0.931) in patients using the smaller surface area membranes (1.5 m^2^ and 1.7 m^2^). The low Qd in patients using the larger surface area membranes (1.8 m^2^ and 2.1 m^2^) resulted in a significant decrease in online Kt/V (1.12 ± 0.25 for 300 Qd and 1.17 ± 0.24 for 500 Qd, p = 0.006) (Figure).

## 4. Discussion

We investigated the effect of reduced dialysate flow rate on dialysis adequacy by using online Kt/V and found three important findings. Firstly, we found no difference in online Kt/V readings between the standard Qd and the low Qd for the overall study population. Secondly, online Kt/V was not different between the standard Qd and the low Qd for women and patients less than 65 kg; however, online Kt/V was reduced with the low Qd in men and patients weighing more than 65 kg. Finally, the low Qd in patients with a dialyzer membrane area over 1.8 m^2^ reduced the dialysis adequacy calculated by online Kt/V.

While increases in Qd produced limited improvements in HD adequacy in most studies, several studies reported that a reduced Qd did not reduce or alter HD clearance. The most important reason for this discrepancy in the literature may have stemmed from inadequacies in patient selection and a lack of detailed subgroup analyses [9–11]. Albalate et al. investigated the effects of low and high Qd on HD adequacy compared to the standard Qd and found that HD adequacy was 4% higher at 500 mL/min compared to 400 mL/min and 3% lower at 500 mL/min compared to 700 mL/min. [9]. However, in contrast to these limited changes, many studies in the literature show that Qd variations do not act on HD adequacy [10,11]. Alayoud et al. compared Kt/V at Qd values of 700 mL/min, 500 mL/min, and 404 mL/min and showed that none of the 3 dialysate flow rates had significant effects on dialysis clearance [10]. Ward et al. reported no significant differences in dialysis clearance when dialysate flow rates of 500 mL/min and 800 mL/min were compared [11]. In our study, online Kt/V was similar in both dialysate flow rates for the overall study population. Importantly, we found significant differences in HD adequacy in the subgroup analyses when patients were grouped by gender, body weight, and type of dialyzer.

Major factors to be considered in chronic HD prescription include body weight and body surface area. A pivotal and interesting finding of our study emerged when we compared the online Kt/V readings between patient subgroups based on mean body weight (64.14 ± 15.50). For patients weighing more than 65 kg, the low Qd resulted in worse online Kt/V readings than the standard Qd, but this was not the case for patients weighing less than 65 kg. Two studies with a similar design in the literature compared the standard Qd and a reduced Qd of 400 mL/min in patients weighing less than 60 kg and 65 kg, respectively, and found that online Kt/V at the low Qd was not worse than that at the standard Qd for these patient groups [12,13]. Underweight patients have less total body water, resulting in a smaller urea distribution volume. Thus, it may be possible to reduce Qd without compromising the effectiveness of dialysis in this patient group. HD at rates lower than the standard Qd in low body weight patients can result in significant water savings without substantial reductions in online Kt/V.

Another point to be considered for individualized HD prescriptions is gender. In our study, the reduction of Qd did not cause a significant change in online Kt/V for women, but the reduction of Qd for men resulted in significant reductions in online Kt/V. To our knowledge, no other study has examined the effect of gender difference on online Kt/V at varying Qd. We suggest that the different effects of Qd in the gender groups in our study occurred because of the differences in total body water, muscle mass, and body weight between genders. Total body water percentage, body surface area, and body weight are, on average, lower in women than in men. Therefore, adequate dialysis clearance can be achieved at a reduced Qd in patients with such characteristics [14].

An essential point to consider in dialysis adequacy is the surface area of the dialyzer membrane used. Membranes of 1.5 m^2^, 1.7 m^2^, 1.8 m^2^, and 2.1 m^2^ were used in our study. Dialysis clearance was better at the standard Qd in patients using the larger surface area membranes compared to other dialyzer membranes with different surface areas. There were no differences in online Kt/V at the 2 different Qd values in HD patients using the smaller surface area membranes. While increased Qd with the use of smaller surface area dialyzers does not cause significant variability in dialysis clearance, it significantly increases dialysis clearance in larger surface area dialyzers [15]. Dialysis clearance increases with increasing blood and dialysate flow rates in membranes with a larger surface area. This may be related to the larger surface areas of the membrane priming volumes for the dialysate. Also, increases in the blood or dialysate flow rates decrease the thickness of the respective stagnant fluid layer, leading to improved dialysis effectiveness [16].

There was no statistically significant difference in the postsession mean arterial pressure (MAP) between the low Qd and standard Qd groups, nor in terms of the change from pre- and postsession MAP. It has already been established that the effect of Qd on blood pressure during and after dialysis is limited [17]. We monitored the volume effectively during and after dialysis, ensuring adequate blood pressure levels. Thus, we can suggest that the reduced Qd does not result in variabilities in the volume and is comparable to the standard Qd in this respect.

Our study had some limitations. We evaluated dialysis adequacy by comparing only online Kt/V values from 2 sets of 3 sessions of HD. Because we did not use other parameters in the assessments, our results may not provide an assessment of the overall HD adequacy. Large-scale studies with increased numbers of HD sessions in patient groups from different ethnic origins are needed for the generalization of study results.

## 5. Conclusion

Individualized HD prescriptions and the use of reduced water volumes by employing different dialysis methods are among the ways that nephrologists can contribute to the fight against depleting water resources. In light of our study results, we suggest that a reduced Qd to 300 mL/min in eligible patients can save 48 L of water per HD session and an average of 7500 L of water per year.

## Figures and Tables

**Figure:**
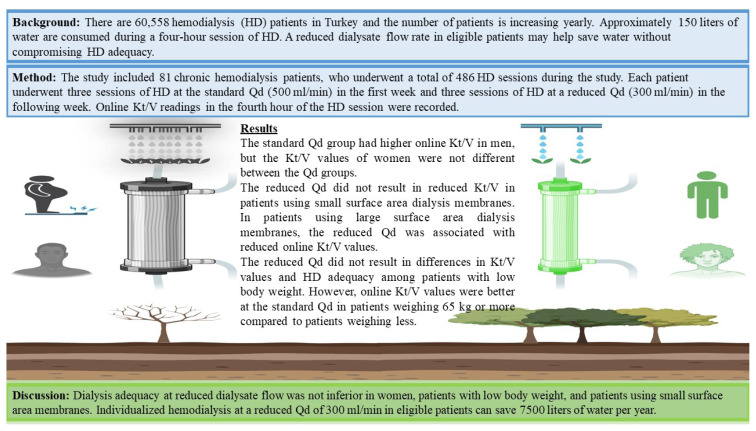
Study brief.

**Table 1:** Demographic and clinical characteristics of patients.

Parameters	n (%) or mean ± SD
Age (years)	49.65 ± 16.64
Gender (women / men)	41 / 40
Weight (kg)	64.14 ± 15.50
Body mass index (kg/m^2^)	23.86 ± 5.17
Diabetes mellitus	24 (29.6%)
Hypertension	51 (63%)
Coronary artery disease	20 (24.7%)
Heart failure	20 (24.7%)
Duration of HD treatment (months)	70.86 ± 63.69
BMI classification (kg/m^2^)	
Underweight (<18.5)	11 (13.6%)
Normal weight (18.5–24.9)	41 (50.6%)
Overweight (25–29.9)	18 (22.2%)
Obese (≥30)	11 (13.6%)
HD access route	
Tunneled intravenous catheter	27 (33.3%)
Arteriovenous fistula	54 (66.7%)
Dialyzer membrane	
1.5 m^2^	18 (22.2%)
1.7 m^2^	30 (37%)
1.8 m^2^	27 (33.3%)
2.1 m^2^	6 (7.4%)

**Table 2:** Dialysis adequacy at standard and reduced dialysate flow rates.

Parameters	Qd (mL/min)	Mean ± SD	t	p
All patients (n = 81)				
Online Kt/V	300	1.49 ± 0.44	−1.952	0.069
	500	1.51 ± 0.41		
Ultrafiltration volume (mL)	300	2208 ± 950	−4.463	0.001
	500	2519 ± 1088		
MAP after HD (mmHg)	300	88 ± 13	0.009	0.993
	500	88 ± 14		
Difference in MAP before and after HD (mmHg)	300	6 ± 11	1.158	0.250
	500	8 ± 11		
Female patients (n = 41)				
Online Kt/V	300	1.68 ± 0.46	0.080	0.937
	500	1.68 ± 0.44		
Ultrafiltration volume (mL)	300	2210 ± 912	3.088	0.002
	500	2500 ± 1043		
MAP after HD (mmHg)	300	84 ± 15	0.801	0.428
	500	82 ± 15		
Difference in MAP before and after HD (mmHg)	300	5 ± 12	1.407	0.160
	500	9 ± 9		
Male patients (n = 40)				
Online Kt/V	300	1.30 ± 0.32	2.345	0.019
	500	1.35 ± 0.30		
Ultrafiltration volume (mL)	300	2206 ± 999	3.019	0.004
	500	2537 ± 1145		
MAP after HD (mmHg)	300	91 ± 9	0.814	0.420
	500	93 ± 11		
Difference in MAP before and after HD (mmHg)	300	7 ± 10	0.136	0.892
	500	7 ± 12		
Patients under 65 Kg (n = 51)				
Online Kt/V	300	1.66 ± 0.41	0.698	0.486
	500	1.67 ± 0.37		
Ultrafiltration volume (mL)	300	2122 ± 859	2.327	0.001
	500	2510 ± 1022		
MA after HD (mmHg)	300	84 ± 13	0.811	0.359
	500	83 ± 11		
Difference in MAP before and after HD (mmHg)	300	6 ± 15	1.097	0.211
	500	8 ± 9		
Patients over 65 Kg (n = 30)				
Online Kt/V	300	1.22 ± 0.34	2.314	0.028
	500	1.25 ± 0.32		
Ultrafiltration volume (mL)	300	2338 ± 1075	2.681	0.002
	500	2523 ± 1197		
MAP after HD (mmHg)	300	92 ± 8	0.799	0.451
	500	93 ± 13		
Difference in MAP before and after HD (mmHg)	300	6 ± 11	0.911	0.367
	500	7 ± 11		
Patients who underwent HD with 1.5 m^2^ and 1.7 m^2^ dialyzer membranes (n = 48)				
Online Kt/V	300	1.75 ± 0.35	0.125	0.901
	500	1.75 ± 0.32		
Ultrafiltration volume (mL)	300	2221 ± 918	3.155	0.008
	500	2516 ± 1024		
MAP after HD (mmHg)	300	86 ± 13	0.721	0.411
	500	83 ± 12		
Difference in MAP before and after HD (mmHg)	300	7 ± 12	1.179	0.343
	500	8 ± 10		
Patients who underwent HD with 1.8 m^2^ and 2.1 m^2^ dialyzer membranes (n = 33)				
Online Kt/V	300	1.12 ± 0.25	2.973	0.006
	500	1.17 ± 0.24		
Ultrafiltration volume (mL)	300	2313 ± 976	2.661	0.012
	500	2577 ± 1083		
MAP after HD (mmHg)	300	91 ± 12	0.119	0.145
	500	89 ± 14		
Difference in MAP before and after HD (mmHg)	300	7 ± 13	1.378	0.417
	500	7 ± 11		

MAP = mean arterial pressure; HD = hemodialysis.
